# RT‐LAMP for rapid diagnosis of coronavirus SARS‐CoV‐2

**DOI:** 10.1111/1751-7915.13586

**Published:** 2020-04-25

**Authors:** Wei E. Huang, Boon Lim, Chia‐Chen Hsu, Dan Xiong, Wei Wu, Yejiong Yu, Huidong Jia, Yun Wang, Yida Zeng, Mengmeng Ji, Hong Chang, Xiuming Zhang, Hui Wang, Zhanfeng Cui

**Affiliations:** ^1^ Oxford Suzhou Centre for Advanced Research (OSCAR) University of Oxford Suzhou Industrial Park Jiangsu China; ^2^ Department of Engineering Science University of Oxford Parks Road OX1 3PJ Oxford UK; ^3^ Institute of Biomedical Engineering Department of Engineering Science University of Oxford Oxford OX3 7DQ UK; ^4^ Medical Laboratory of Shenzhen Luohu People's Hospital Shenzhen 518001 China

## Abstract

The pandemic coronavirus SARS‐CoV‐2 in the world has caused a large infected population suffering from COVID‐19. To curb the spreading of the virus, WHO urgently demanded an extension of screening and testing; thus, a rapid and simple diagnostic method is needed. We applied a reverse transcription‐loop‐mediated isothermal amplification (RT‐LAMP) to achieve the detection of SARS‐CoV‐2 in 30 min. We designed four sets of LAMP primers (6 primers in each set), targeting the viral RNA of SARS‐CoV‐2 in the regions of orf1ab, S gene and N gene. A colorimetric change was used to report the results, which enables the outcome of viral RNA amplification to be read by the naked eye without the need of expensive or dedicated instrument. The sensitivity can be 80 copies of viral RNA per ml in a sample. We validated the RT‐LAMP method in a hospital in China, employing 16 clinic samples with 8 positives and 8 negatives. The testing results are consistent with the conventional RT‐qPCR. In addition, we also show that one‐step process without RNA extraction is feasible to achieve RNA amplification directly from a sample. This rapid, simple and sensitive RT‐LAMP method paves a way for a large screening at public domain and hospitals, particularly regional hospitals and medical centres in rural areas.

## Graphical abstract

A rapid method for the detection of SARS‐CoV‐2 RNA virus.

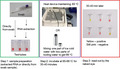

## Introduction

There is an urgent need for rapid diagnosis of SARS‐CoV‐2 infected COVID‐19 patients even before an immune response can occur and for asymptomatic carriers. This is critical in making decisions on public health measures, such as movement restrictions, and quarantine duration. Nucleic acid testing, that is detecting the viral RNA, is a feasible and practical method. Currently, the most common method for nucleic acid diagnosis is based on real time RT‐PCR (Drosten *et al.*, 2002; Mackay *et al.*, 2002; Espy *et al.*, 2006). For example, BGI in China (https://www.bgi.com/kit) and the US CDC (https://www.cdc.gov/coronavirus/2019-ncov/about/testing.html) provide reagents, primers and probes to support RT‐PCR diagnosis for SARS‐CoV‐2. Although real‐time RT‐PCR is sensitive and reliable, it is time‐consuming (~2 h) and requires a specific detection device or instrument, which limits its broad application to current huge demand for the global pandemic of COVID‐19.

To address this challenge, a fast and simple‐to‐operate test kit would be highly desirable. With such a test, virus infected patients could be identified at an early stage and quarantined to prevent the spread of the infection, whilst the non‐infected individuals could carry on with their life as usual. Ideally, the test kit is mobile without the need of any complicated instrument and the test result can be read by the naked eye. Hence, it can be used at airports, railway stations and hospitals, particularly regional hospitals and medical centres in rural areas.

Loop‐mediated isothermal amplification (LAMP) is a rapid technology of DNA amplification (Notomi *et al.*, 2000; Tomita *et al.*, 2008), which has been applied to pathogen detection such as virus, bacteria and malaria (Thai *et al.*, 2004; Boehme *et al.*, 2007; Mori and Notomi, 2009; Law *et al.*, 2015; Reboud *et al.*, 2019). The LAMP reaction generally takes place in a constant temperature, and the target DNA can be amplified in 30 min (Tomita *et al.*, 2008). The LAMP method employs 4 or 6 primers to bind six regions of a target DNA, and the specificity is extremely high (Notomi *et al.*, 2000; Tomita *et al.*, 2008). Since the LAMP method only needs one constant temperature (usually 65°C), the device can be simple. Initially, the LAMP uses 4 primers (Notomi *et al.*, 2000; Tomita *et al.*, 2008), later it is found that the addition of two loop primers can shorten half of the time required for the original LAMP reaction (Nagamine *et al.*, 2002). The availability of WarmStart RTx Reverse Transcriptase (New England Biolabs, UK) makes it possible to combine both reverse transcription and LAMP in one reaction. Since SARS‐CoV‐2 is a RNA virus with the length about 30 kb (Wang *et al.*, 2020), the single reaction of reverse transcription (RT) and LAMP together can significantly shorten the reaction time without the DNA purification step from RT, thus a rapid detection of SARS‐CoV‐2 can be achieved.

In this work, we developed a COVID‐19 diagnosis kit for the rapid detection of SARS‐CoV‐2, using one‐step reverse transcription and loop‐mediated isothermal amplification (RT‐LAMP). The whole reaction can be as short as 20 min at a constant 65°C. The detection limit is 80 copies of viral RNA per ml sample. A simple colour change indication can be visualized by the naked eye to confirm the result of viral RNA amplification. The kits were validated by 16 clinical COVID‐19 samples. This new technique can also provide a rapid and feasible approach for the detection of various virus.

## Results

### Specificity of O117, S17, N1 and N15 primers to amplify synthesized DNA of SARS‐CoV‐2

We designed four sets of LAMP primers O117, S17, N1 and N15, which target RNA encoding Orf1ab, spike glycoprotein and two regions in nucleocapsid protein of SARS‐CoV‐2 respectively (Fig. S1). Each set has 6 primers (Table 1). SARS‐CoV‐2 is a single stranded RNA virus with the length of about 30 kb. Orf1ab is about 21kb long, encoding the replicase polyprotein (Woo *et al.*, 2010). O117 primers were designed to cover the conversed region of 5’‐end of the viral RNA in Orf1ab (Fig. S1). S17 primers target the S gene, which encodes spike glycoprotein, a key for this SARS‐CoV‐2 virus to bind human ACE2 protein and to invade human cells (Menachery *et al.*, 2015; Cui *et al.*, 2019; Walls *et al.*, 2020). The N gene for nucleocapsid protein is at the 3ʹ‐end of the viral RNA, conserved to SARS‐like coronavirus (Cui *et al.*, 2019). During sampling and RNA extraction processes, the viral RNA might be attacked by RNase, degrading from 5ʹ‐ to 3ʹ‐, so we designed N1 and N15 to ensure the detection of 3ʹ‐end of the viral RNA of SARS‐CoV‐2, even though it is partially degraded. The targeted SARS‐CoV‐2 RNA fragments of these four sets of primers (Fig. S1 and Table 1) are about 240–260 bp, long enough to be targeted by 6 primers and short enough to achieve a rapid amplification.

**Table 1:** Primers for the detection of SARS‐CoV‐2.

N1	Sequence 5ʹ‐3ʹ
F3	TGGACCCCAAAATCAGCG
B3	GCCTTGTCCTCGAGGGAAT
FIP^a^	CCACTGCGTTCTCCATTCTGGTAAATGCACCCCGCATTACG
BIP	CGCGATCAAAACAACGTCGGCCCTTGCCATGTTGAGTGAGA
LF	TGAATCTGAGGGTCCACCAAA
LB	GGTTTACCCAATAATACTGCGTCTT

N15	Sequence 5ʹ‐3ʹ
F3	AGATCACATTGGCACCCG
B3	CCATTGCCAGCCATTCTAGC
FIP^a^	TGCTCCCTTCTGCGTAGAAGCCAATGCTGCAATCGTGCTAC
BIP	GGCGGCAGTCAAGCCTCTTCCCTACTGCTGCCTGGAGTT
LF	GCAATGTTGTTCCTTGAGGAAGTT
LB	GTTCCTCATCACGTAGTCGCAACA

S17	Sequence 5ʹ‐3ʹ
F3	TCTTTCACACGTGGTGTT
B3	GTACCAAAAATCCAGCCTC
FIP^a^	CATGGAACCAAGTAACATTGGAAAACCTGACAAAGTTTTCAGATCC
BIP	CTCTGGGACCAATGGTACTAAGAGGACTTCTCAGTGGAAGCA
LF	GAAAGGTAAGAACAAGTCCTGAGT
LB	CTGTCCTACCATTTAATGATGGTGT

O117	Sequence 5ʹ‐3ʹ
F3	CCCCAAAATGCTGTTGTT
B3	TAGCACGTGGAACCCAAT
FIP^a^	GGTTTTCAAGCCAGATTCATTATGGATGTCACAATTCAGAAGTAGGA
BIP	TCTTCGTAAGGGTGGTCGCAGCACACTTGTTATGGCAAC
LF	TCGGCAAGACTATGCTCAGG
LB	TTGCCTTTGGAGGCTGTGT

Human β‐actin primers (Poon *et al.*, 2005)	
β‐actin	Sequence 5ʹ‐3ʹ
F3	ACTGGGACGACATGGAGAA
B3	TCACCGGAGTCCATCACG
FIP	GTTGGCCTTGGGGTTCAGGGTTCTACAATGAGCTGCGTGT
BIP	TTGAGACCTTCAACACCCCAGCGAGGCGTACAGGGATAGCA

^a^ The 5ʹ‐ of FIP primer was labelled by FAM for the application to hospital.

In all experiments of LAMP and RT‐LAMP, the experiments were carried out with more than three biological replicates and the results were consistent.

**Fig. 1:**
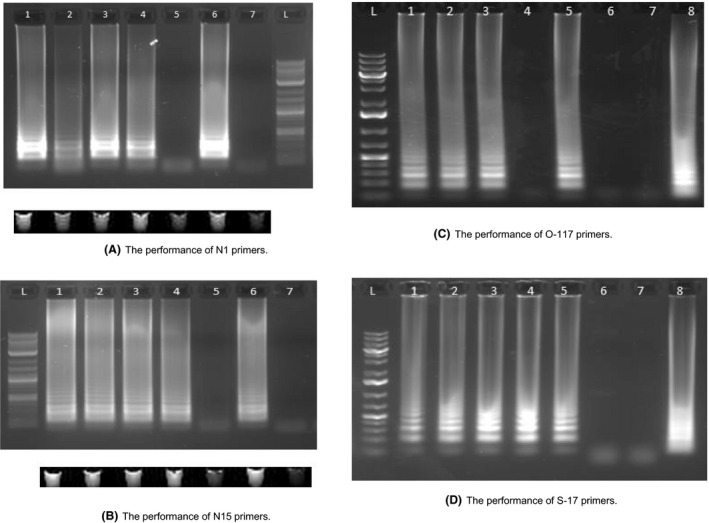
LAMP results of four different sets of primers targeting the DNA of N, S and Orf1ab gene of SARS‐CoV‐2. A. Performance of N‐1 primer set using N gene DNA sequence. (1) N‐15 primers + N gene DNA (200k copies)*; (2) N15 primers + N gene DNA (200 copies); (3) N15 primers + N gene DNA (200k copies) + Human genome; (4) N15 primers + N gene DNA (200 copies) + Human genome; (5) N15 primers + Human genome; (6) Human β‐actin primers + Human genome; (7) Human β‐actin primers + N gene DNA (200k copies); L, Ladder. LAMP results using fluorescent dye were visualized under UV exposure, which is consistent to the result of gel electrophoresis. B. Performance of N‐15 primer set using N gene DNA sequence. (1) N‐15 primers + N gene DNA (200k copies); (2) N15 primers + N gene DNA (200 copies); (3) N15 primers + N gene DNA (200k copies) + Human genome; (4) N15 primers + N gene DNA (200 copies) + Human genome; (5) N15 primers + Human genome; (6) Human β‐actin primers + Human genome; (7) Human β‐actin primers + N gene DNA (200k copies); L, Ladder. LAMP results using fluorescent dye were visualized under UV exposure, which is consistent to the result of gel electrophoresis. C. Performance of O‐117 primer set using Orf1ab gene DNA sequence. (1) O‐117 primers + Orf1ab gene DNA (200k copies); (2) O‐117 primers + Orf1ab gene DNA (200 copies); (3) O‐117 primers + Orf1ab gene DNA (20 copies); (4) O‐117 primers + Orf1ab gene DNA (2 copies); (5) O‐117 Primers + Orf1ab gene DNA (200 copies) + Human Genome; (6) O‐117 Primers + Human Genome; (7) O‐117 Primers + water (8) Human β‐actin primers + human genomic DNA; (L) Ladder. D. Performance of S‐17 primer set using S gene DNA sequence. (1) S‐17 primers + S gene DNA (200k copies); (2) S‐17 primers + S gene DNA (200 copies); (3) S‐17 primers + S gene DNA (20 copies); (4) S‐17 primers + S gene DNA (2 copies); (5) S‐17 Primers + S gene DNA (200 copies) + Human Genome; (6) S‐17 Primers + Human Genome; (7) S‐17 primers + water (8) Human β‐actin primers + human genomic DNA; (L) Ladder. *200k (200,000) 200, 20 and 2 represent the total copy number of DNA sequence in the reaction mix.

To verify their specificity, we first tested these primers using synthetic target DNA of SARS‐CoV‐2. N1 and N15 primers were able to amplify DNA fragments of the N gene, but not human genomic DNA (Fig. 1A and B). By adding the fluorescent dye provided by NEB (New England Biolabs, UK), positive results can be visualized directly from the PCR tubes under UV exposure. The fluorescent intensity in the amplification‐positive tubes was stronger than that in the amplification‐negative tubes, although fluorescence background was also present in the negative tubes (Fig. 1A and B). The fluorescent read‐outs are consistent to the results by electrophoresis gel. However, the fluorescent signal background might cause ambiguous reading and it requires fluorescent sensitive instrument to analyse the result. So we changed it to colorimetric reading later in this study.

Similarly, O117 and S17 primers can also specifically amplify the synthesized DNA fragments of Orf1ab gene and S gene, respectively, but they were unable to amplify human genomic DNA (Fig. 1C and D). The addition of human genomic DNA to the viral DNA in the reaction mixture did not interfere with the amplification performance (lane 4 in Fig. 1A and B, lane 5 in Fig. 1C and D), which is essential for using RT‐LAMP to detect viral RNA in a human sample. The LAMP reaction time for all experiments was 30 min. Positive amplification products were obtained even for 2 copies of the synthetic viral DNA fragment template in 30 min when using the S17 primers (lane 4 in Fig. 1D), demonstrating that the LAMP reaction was rapid and sensitive.

### RT‐LAMP and sensitivity to amplify synthesized RNA of SARS‐CoV‐2

**Fig. 2:**
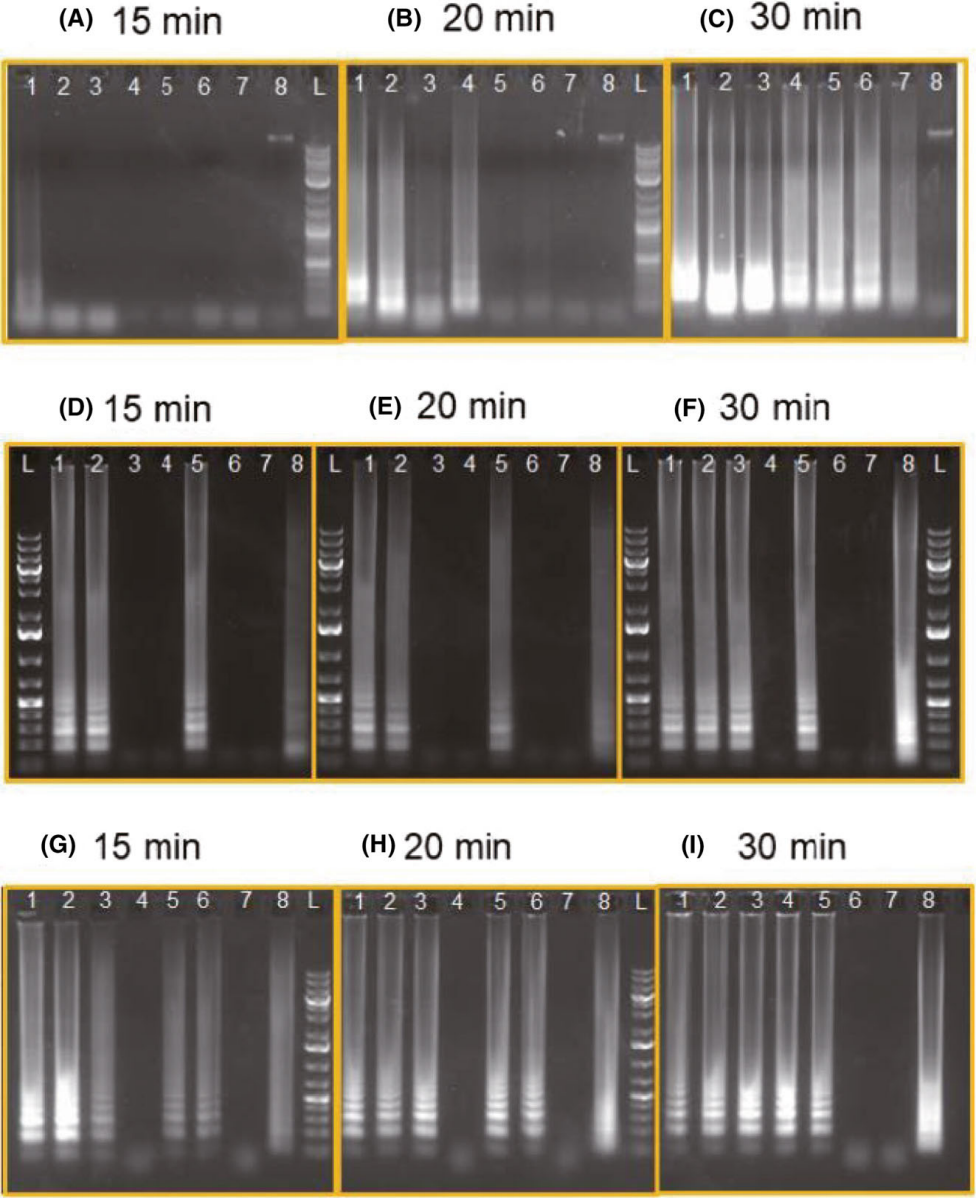
Sensitivity and efficiency of RT‐LAMP using N1 and N15 primers (A, B, C). RNA sequence of N gene was derived from *in vitro* transcription and used as the target. Reaction mixture was sampling at (A) 15 min, (B) 20 min and (C) 30 min. (1) N1 Primers + N gene RNA (200); (2) N1 Primers + N gene RNA (20); (3) N1 Primers + N gene RNA (2); (4) N15 Primers + N gene RNA (200); (5) N15 Primers + N gene RNA (20); (6) N15 Primers + N gene RNA (2); (L) Ladder. Sensitivity and efficiency of RT‐LAMP using O‐117 (D, E, F) and S‐17 primers (G, H, I). RNA of orf1ab and S17 was derived from *in vitro* transcription and used as the targets. Reaction mixture was sampling at (D, G) 15 min, (E, H) 20 min and (F, I) 30 min. (1) Viral Primers + RNA target (200 k); (2) Viral Primers + RNA target (200); (3) Viral Primers + RNA target (20); (4) Viral Primers + RNA target (2); (5) Viral Primers + RNA target (200) + human genome; (6) Viral Primers + human genome; (7) Viral Primers + Water; (8) Human Primers + human genome

To assess the potential of RT‐LAMP in detecting RNA virus of SARS‐CoV‐2, we then tested the performance of these primers with synthesized RNA fragments of the N gene, S gene and Orf1ab gene obtained from *in vitro* transcription (Appendix S1). It shows that reverse transcription and LAMP can be integrated into one single RT‐LAMP reaction to specifically amplify viral RNA fragments (Fig. 2).

We evaluated the sensitivity of RT‐LAMP by diluting the copy number of RNA target from 200 to 2 copies per reaction (in total 25 μl reaction solution) and examined the efficiency by sampling the reaction mixture at 15, 20 and 30 min after the reaction started. It shows that 2 copies of target RNA can be amplified to detectable level using N1 primers within 20 min, whilst it requires 30 min using N15 primers (Fig. 2A–C). The sensitivity and efficiency of RT‐LAMP were also tested on S17 and O117 primers. As shown in Figure 2, 2 copies of S gene RNA can be detected using S17 primers in 30 min, whilst the detection limit using O117 primers is 20 copies of Orf1ab gene RNA in 30 min. The results suggest that RT‐LAMP is sensitive enough to detect viral RNA within a 30‐min reaction and the sensitivity can be 2 copies in 25 μl reaction (80 copies viral RNA per ml) by employing primers N1 and S17.

It is also observed that the higher copies of RNA, the shorter time is needed to obtain the products (Fig. 2). When the copy number of RNA is 200 per reaction, the amplification result can be observed as short as 15 min (Fig. 2).

### Colour change of LAMP reaction mixture provides semi‐quantitative results

**Fig. 3:**
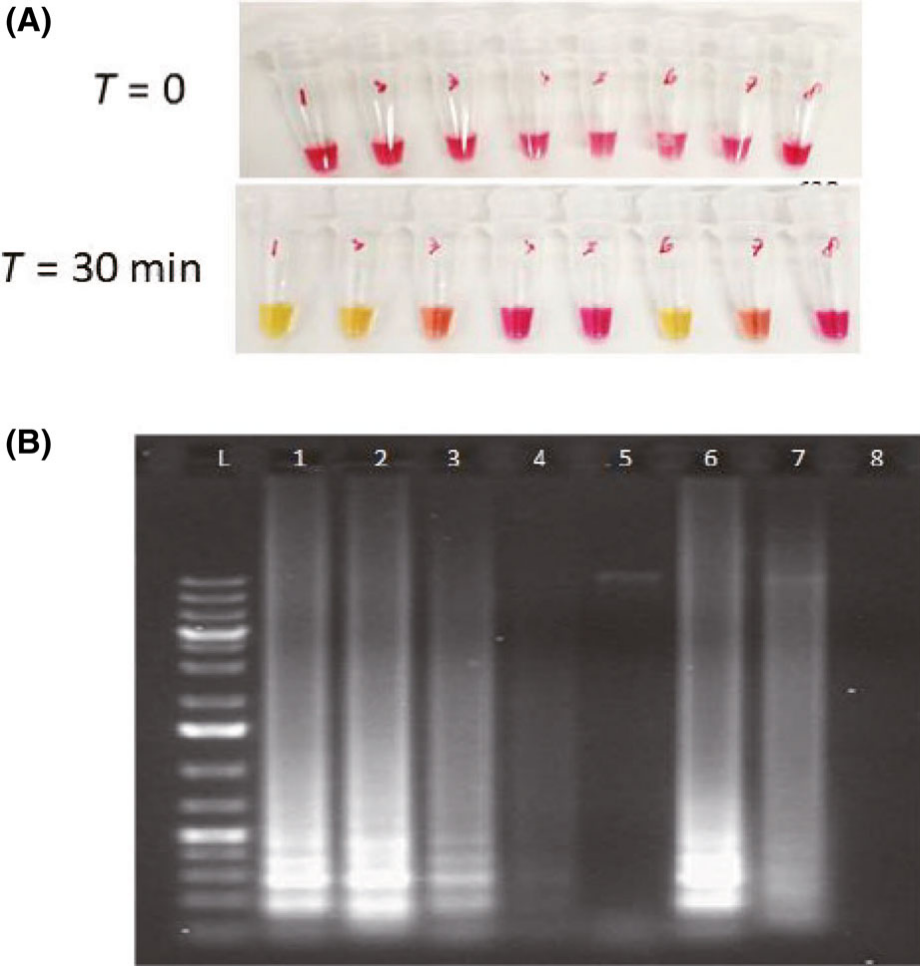
Colorimetric RT‐LAMP assay using N15 primers. A. Colour of reaction mixtures before RT‐LAMP. B. Colour of reaction mixtures after RT‐LAMP. C. Gel electrophoresis of reaction mixtures. The intensity of RT‐LAMP products in the gel tallies with the colour of reaction mixtures and provides a semi‐quantitative results. (1) N15 Primers + N gene RNA (200k); (2) N15 Primers + N gene RNA (200); (3) N15 Primers + N gene RNA (20); (4) N15 Primers + N gene RNA (2); (5) N15 Primers + human genome; (6) Human primers + human genome; (7) Human Primers whole human RNA; (8) Colorimetric MasterMix only.

Since result‐reporting using fluorescent dye requires a specific instrument (Fig. 1), we also tried to read the results using the naked eye. Since the nucleic acid amplification releases pyrophosphate and hydrogen ion, which decreases the pH of reaction solution, a sensitive pH indicator can be used to show the positive or negative result of RT‐LAMP (Tanner *et al.*, 2015; Poole *et al.*, 2017). Hence, RT‐LAMP result can be visualized by a pH indicator such as phenol red, which shows pink 8.2–8.6 and becomes yellow when pH drops. Using the WarmStart™ Colorimetric LAMP 2× Master Mix (DNA & RNA) from New England Biolabs, a visible colour change from pink to yellow could be observed if the amplification of the target sequence has taken place (Fig. 3). We show that the colorimetric RT‐LAMP assay can reliably detect N genes of SARS‐CoV‐2 viral RNA, and the colour change could indicate the number of target sequence semi‐quantitatively (Fig. 3).

### Both colorimetric and fluorescent reading in one RT‐LAMP reaction

**Fig. 4:**
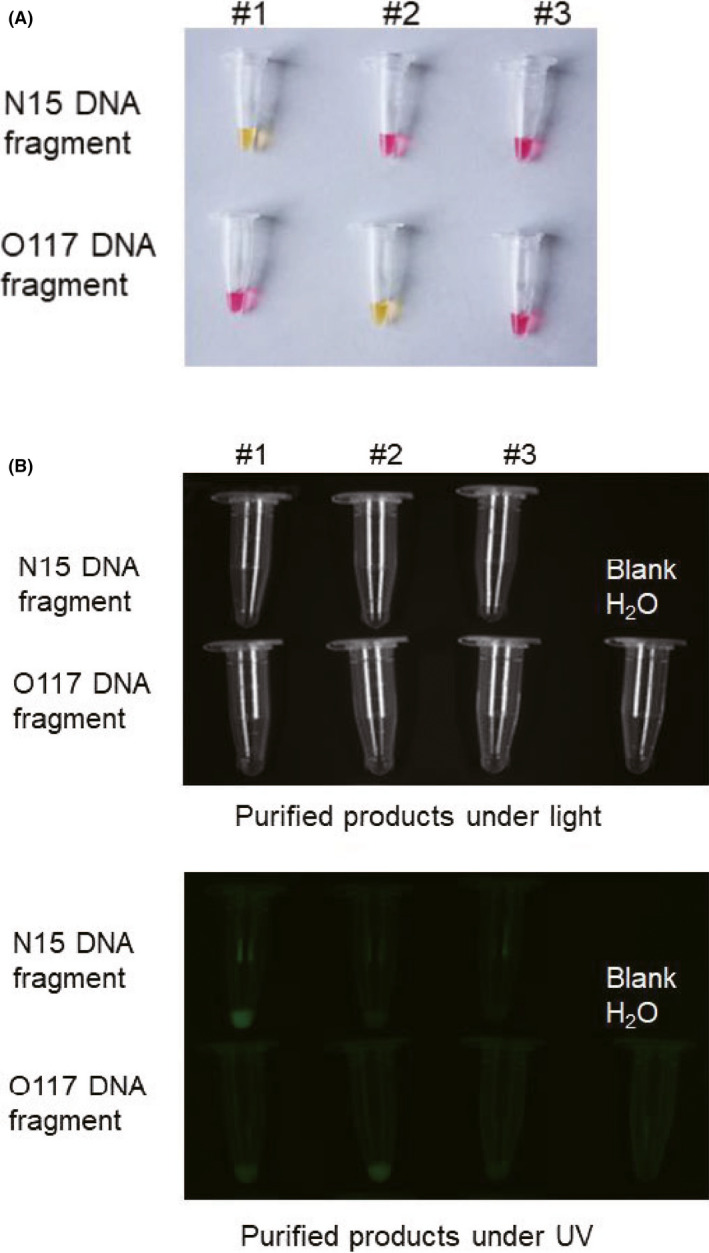
The products of RT‐LAMP can be read by both colour change and fluorescent signal, which are consistent. A. Colorimetric display of the RT‐LAMP products. B. Fluorescent image under the UV lamp of the RT‐LAMP products. #1, #2 and #3 samples separately contained N15, O117 and β‐actin primers.

Colorimetric reading is dependent on pH change during RT‐LAMP. However, in clinical application, many factors might interfere pH change. For example, patient sample conditions can be different, introducing uncertain factors to pH change in the RT‐LAMP reaction. In addition, viral RNA of SARS‐CoV‐2 might be eluted by various buffers, which might be compatible to RT‐LAMP reaction but could significantly affect the colorimetric reading (Fig. S2). To overcome the uncertainty and ensure the reliability of the diagnosis results, we also designed another set of FIP primers, which 5ʹ‐end was conjugated with fluorescent FAM (6‐Carboxyfluorescein; Table 1). Figure 4 shows that the application of 5ʹ‐FAM‐FIP primers was able to display both colorimetric and fluorescent readings, and both results were consistent.

### Clinical test validates the performance of RT‐LAMP

**Fig. 5:**
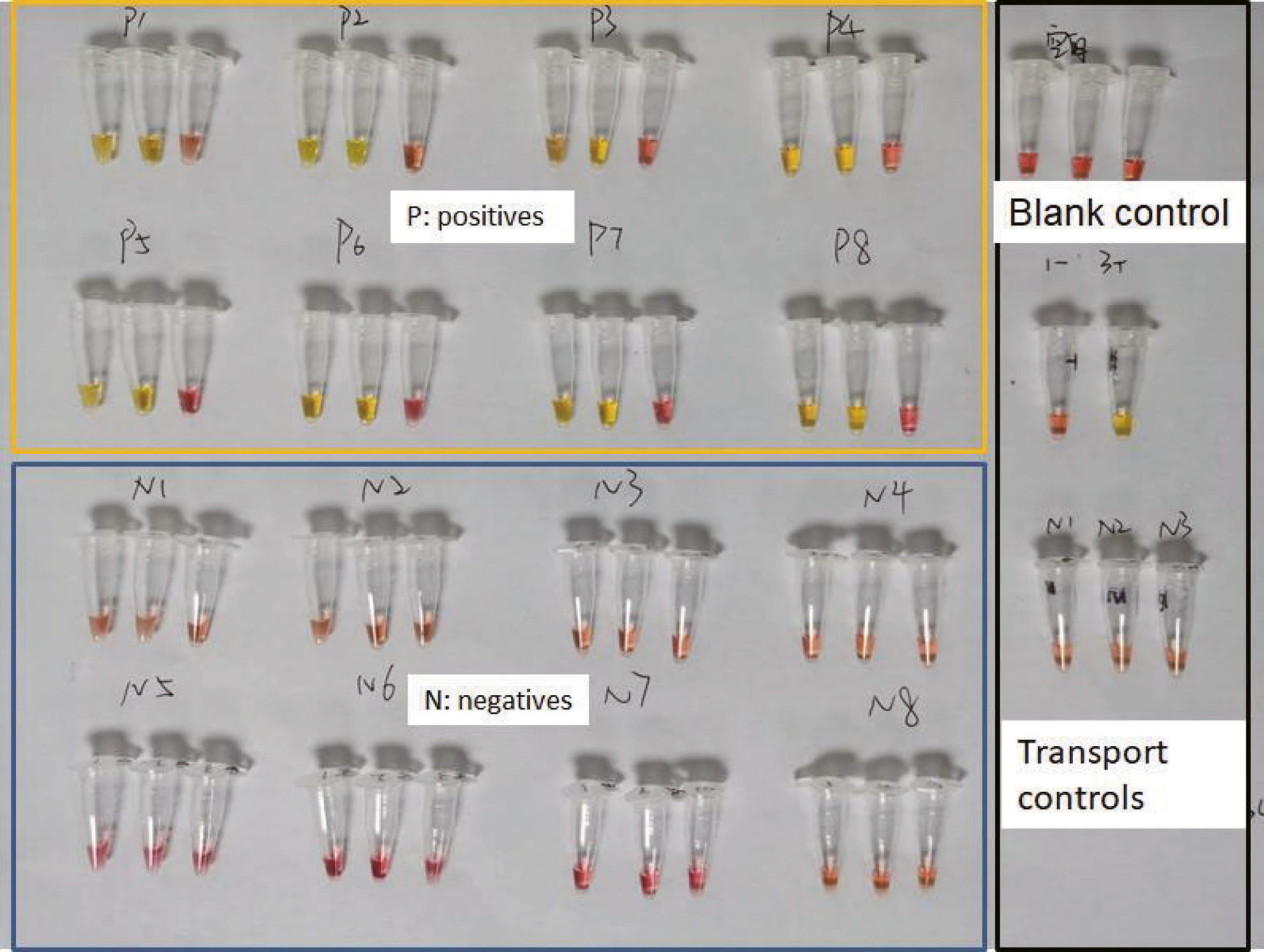
Validation of RT‐LAMP using 16 clinical COVID‐19 samples from Shenzhen Luohu People’s hospital. Samples were first tested with conventional RT‐PCR (Table 2), and eight positive samples were labelled ‘P’, and eight negative samples were labelled ‘N’. The components of each test kit are listed in Experimental procedures. Three tubes in each RT‐LAMP assay are #1, #2, #3 in order from left to right. Tube #1 and #2 separately contain O117 and N15 primers targeting the Orf1ab gene and N gene of SARS‐CoV‐2. Tube #3 contain human β‐actin primers. To tube #1 and 2, all positive samples turned yellow, whilst all negative samples remained pink after 30‐minute reaction. The results of RT‐LAMP method are consistent to the results of conventional RT‐PCR (Table 2 and Fig. S1).

To validate the performance of RT‐LAMP in diagnosing COVID‐19, a test kit consisting of three tubes (#1: O117 primers, #2: N15 primers, #3: human β‐actin primers; See Materials and Methods) was sent to Shenzhen Luohu People’s hospital for clinical validation. RT‐qPCR and RT‐LAMP tested the 16 clinical samples (8 positive and 8 negative samples) in parallel, which were taken by swab from patients in a standard way. The samples were initially tested by conventional RT‐qPCR; then, RT‐LAMP was used to test the same samples. Using RT‐LAMP, tubes #1 and #2 of each testing kit turned yellow which indicate the presence of target viral RNA in all 8 positive samples, whilst tube #1 and #2 remained pink for all 8 negative samples (Figure 5). This shows the RT‐LAMP assay has a good agreement with the conventional RT‐PCR results in diagnosing COVID‐19 samples (Fig. 5 and Fig. S3). Tube #3 of all test kits remained pink in both positive and negative samples, which indicates the human β‐actin primers are not able to detect the β*‐actin* gene transcripts within the patient samples. Comparing the results between RT‐qPCR and RT‐LAMP, it shows that the RT‐LAMP was able to detect viral RNA with *C_t_* = 36 by RT‐qPCR (Table 2), suggesting that RT‐LAMP with visually colour change reading is very sensitive.

**Table 2:** Comparison of conventional RT‐qPCR and LAMP results for clinical samples.

Sample number	RT‐qPCR result (*Ct* value)				LAMP result	
	ORF1ab	N	E	IC	N‐15	O‐117
P1	35.78	34.84	34.9	25.94	+	+
P2	31.14	29.24	28.87	25.86	+	+
P3	34.48	32.13	30.37	25.84	+	+
P4	36.64	34.91	31.18	26.15	+	+
P5	31.34	31.11	27.53	25.7	+	+
P6	31.61	30.49	26.92	25.88	+	+
P7	29.93	20.98	25.51	26.07	+	+
P8	33.15	33.09	31.55	28.95	+	+
N1	–	–	–	26.33	−	−
N2	–	–	–	25.89	−	−
N3	–	–	–	26.65	−	−
N4	–	–	–	25.04	−	−
N5	–	–	–	26.31	−	−
N6	–	–	–	26.56	−	−
N7	–	–	–	26.89	−	−
N8	–	–	–	26.11	−	−

Ct, cycle threshold; E, E gene for envelop protein; IC, RNase gene as internal control; N, N gene for nucleocapsid protein; ORF1ab, orf1ab gene.

### One‐step process of nucleic acid detection using RT‐LAMP

**Fig. 6:**
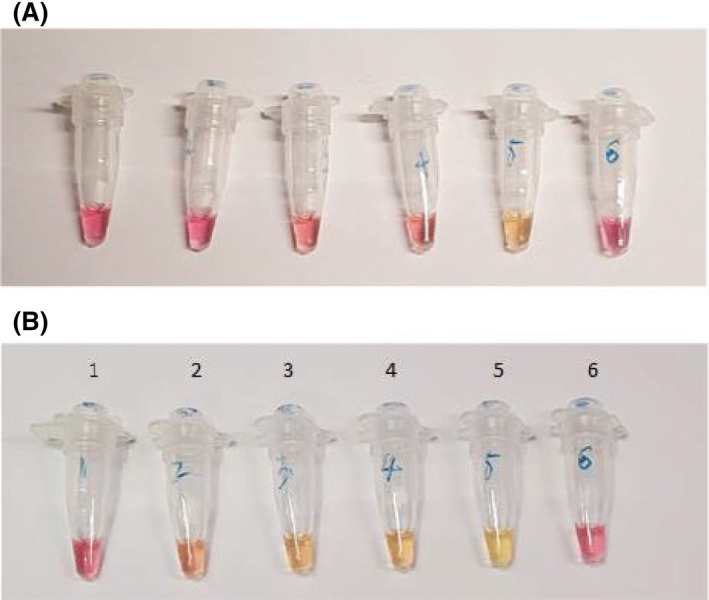
One‐step process to get the products of nucleic acid amplification. One microlitre of iPSC cells in 0.85% sterile saline was directly added into the LAMP reagents in each tube and incubate for 30 min (A) and 40 min (B). The whole process was 40 min. (1) 0 cells + Human β‐actin primers. (2) 10 cells + Human β‐actin primers. (3) 50 cells + Human β‐actin primers. (4) 100 cells + Human β‐actin primers. (5) Extracted human RNA + Human β‐actin primers. (6) H_2_O + Human β‐actin primers.

Cell samples can be processed in one single step to obtain nucleic acid amplification. Heating cells at the same temperature of RT‐LAMP 65°C should lyse cells or virus and release RNA. In this study, we tested the one‐step process using human Episomal induced pluripotent stem cells (iPSCs), which were suspended in 0.85% saline. Figure 6 shows that the nucleic acids of β‐actin gene in iPSC cells can be directly amplified using RT‐LAMP within 40 min. After 30 min, only the purified human RNA can be amplified, showing the colour change (Fig. 6A). However, after 40 min, the tube with human cells changed the colour, whilst the tube without human cells and blank water control remained unchanged pink colour (Fig. 6B). The one‐step process is sensitive enough to detect human 10 cells per 25‐μl reaction. This one‐step process took 10 min longer than RT‐LAMP to RNA, because it takes about 5–10 min time to lyse cells and release nucleic acids. The result suggests that it should be possible to use one single step to achieve the detection of specific nucleic acids in cells. Further test is needed to validate its practical feasibility on clinical swab samples containing SARS‐CoV‐2.

## Discussion

Here, we described a fast and simple‐to‐operate method to diagnose COVID‐19. We applied RT‐LAMP to achieve a rapid testing of SARS‐CoV‐2, using different primers (O117, S17, N1 and N15) to target Orf1ab gene, S gene and N gene. We showed that a 30 min reaction could detect down to 2 copies of target RNA per 25 μl reaction (80 copies per ml; Figs 1 and 2). However, this extreme sensitivity is also a double‐edged sword: carry‐over contamination was common in LAMP reactions, which usually cause false positive results (Hsieh *et al.*, 2014; Ma *et al.*, 2017). We also observed carry‐over contamination after the RT‐LAMP reactions were performed several times in the laboratory. We suspect the aerosol formed from the RT‐LAMP products were able to spread everywhere in the laboratory, contaminating lab‐coats, pipettes, reagents, materials and equipment used for the next reaction, thus for clinical use all components required in the RT‐LAMP reaction need to be prepared in a separate laboratory space with good practice of molecular biology.

The colorimetric display of the testing outcome is easy without the need of expensive or complicated instrument. However, the colour change of RT‐LAMP reaction is based on pH indicator phenol red (Tanner *et al.*, 2015; Poole *et al.*, 2017). The elution buffers of the various RNA extraction kits will significantly affect the result. Hence, in all of our experiments including the clinical tests, the RNA samples were eluted in DNase/RNase‐free water to ensure the RNA elution buffer has a minimal impact of pH indication in RT‐LAMP reactions. The viral RNA clinical tests work well to match the results of RT‐qPCR. To tube #3, all samples were negative, suggesting that RNA of human β*‐actin* gene transcript was not amplified in the samples (Fig. 5). The four‐primer design for human β*‐actin* (Poon *et al.*, 2005) might require a longer reaction time than 30 min in RT‐LAMP assay. In the future, the better human control should be applied. For example, the primers for human RNase gene can be used as a positive control.

The ultimate aim is to develop an enclosed device that integrates RNA extraction, purification, reverse transcription (RT) and loop‐mediated isothermal amplification (LAMP) to detect the SARS‐CoV‐2 virus directly from a throat swab sample. It has been well‐known that colony PCR can be done directly using cells (Huang *et al.*, 2005). Heating cells can cause cell lysis and nucleic acid release, which can be used as template for the amplification of nucleic acids. We tested one‐step RT‐LAMP for nucleic acid amplification and it worked well (Fig. 6). The advantage is that the same temperature 65 °C for RT‐LAMP was able to lyse cells and the cell lysis in pure water (or with low salt) did not affect the reaction of RT‐LAMP. This preliminary work suggests that it is possible to combine RNA extraction, RT and LAMP in one single step, which will significantly simplify the detection process and shorten the detection time. Current criteria of the test in China are 300 copies ml^−1^; the preliminary results of one‐step RT‐LAMP should meet this requirement.

Proving the practicability of RT‐LAMP in diagnosing COVID‐19 is the first step towards developing a more accessible diagnostic kit. Testing is crucial not just for getting treatment to those in need, but also to curb the spread of disease within a region. As we face a global shortage of testing kits amid this global pandemic, our work will be vital to address this need in clinical practice as well as to extend towards a home‐based or personal diagnostic system.

With this proof‐of‐concept work, it is possible to develop a simple, low cost and one‐step testing kit which can be used to screen individuals for SARS‐CoV‐2 virus at the point‐of‐care testing (POCT). There is a need for an on‐site rapid test for the infection which can be operated easily with minimal training and without the risk of getting infected. Existing methods to detect SARS‐CoV‐2 are either based on detecting the virus RNA itself using reverse transcription real‐time polymerase chain reaction (RT‐qPCR) technologies, or based on specific IgM and IgG from patients' blood generated several days after the infection. Currently, these tests would require a laboratory with specific machines operated by skilled scientists and technicians that take at least 2 h to perform. The ultimate goal will be development of a rapid (30–45 min) enclosed test device with one‐step operation for the detection of SARS‐CoV‐2 virus. Multiple sites on the viral RNA are detected to ensure specificity and sensitivity, and an internal control is embedded to prevent false positives and false negatives. This study will pave the way to move forward for rapid and large‐scale screening and diagnosis, where patients exhibiting suspected symptoms can all be tested in the early stages.

## Experimental procedures

### Cell lines and clinical samples

Human Episomal iPSC Line was purchased from Thermo Fisher (UK) and used as the source of human genomic DNA and RNA.

Shenzhen Luohu People’s hospital is authorized by Chinese CDC for the detection of clinical samples for SARS‐CoV‐2 virus. All clinical samples were sampled by clinical throat swab, stored and transported by virus transport media (VTM). Virus deactivation steps were done following the standard guidelines for detection of nucleic acid of SARS‐CoV‐2 in clinical samples. The research on rapid diagnostic technique for COVID‐19 using clinical samples has been approved by the ethical committee at Shenzhen Luohu People’s hospital.

### Design of primers and DNA/RNA synthesis for RT‐LAMP

Four sets of LAMP primers were designed targeting the Orf1ab, S and N genes of SARS‐CoV‐2 published at NCBI (GenBank NC_045512.2). Primers were designed using LAMP primer designing software, PrimerExplorer (http://primerexplorer.jp/e/; Tomita *et al.*, 2008). six primers including forward primer F3, backward primer B3, forward inner primer FIP, backward inner primer BIP, forward loop primer LF and backward loop primer LB were designed to accelerate the LAMP reaction (Nagamine *et al.*, 2002). Table 1 shows all the primer sequences used in this work. In some experiments, 5ʹ‐end of FIP primers were labelled by FAM. To carry out mock experiment, we also designed four DNA fragments, each containing a T7 promoter for in vitro transcription (Appendix S1). All designed primers and DNA fragments, as well as a 2019‐nCoV_N_Positive Control plasmid, were ordered from Integrated DNA Technologies (IDT, UK).

### DNA and RNA purification

The DNA fragments of N, S and Orf1ab target genes were synthesized by IDT and reconstituted to 50 ng µl^−1^ according to the manufacturer’s instruction. The copy number of each target gene was determined from their molecular weight and diluted into 200 000, 200, 20 and 2 copies µl^−1^ for subsequent experiments.

T7_N, T7_S, and T7_O DNA synthesized by IDT were reconstituted to 50 ng µl^−1^ according to the manufacturer’s instruction. These DNA sequences were subjected to in vitro transcription using HiScribe™ T7 High Yield RNA Synthesis Kit (New England Biolabs, UK). Transcription products were purified using RNeasy Mini Kit (Qiagen, UK), and the concentration and quality of RNA were measured using Nanodrop. The copy number of each target RNA was determined from their molecular weight and diluted into 200 000, 200, 20 and 2 copies µl^−1^ for subsequent experiments.

Human whole‐genome was purified from human iPSC cells using Fast DNA™ SPIN Kit from MP Biomedicals followed by followed by DNA Cleanup Kit (New England Biolabs, UK). Human whole‐RNA was purified from human iPSCs using RNeasy Mini Kit (Qiagen, UK).

### LAMP and RT‐LAMP for SARS‐CoV‐2

All equipment, laminar‐flow cabinet and working bench were sprayed with RNaseZap™ prior to experimental work. Filtered pipette tips were used to prevent cross‐contamination. All experiments of LAMP and RT‐LAMP were run for at least three replicates.

A 10X primer mix (FIP, 16 µM; BIP, 16 µM; F3, 2 µM; B3, 2 µM; LF, 4 µM; LB, 4 µM) was made before the reactions. Both LAMP and RT‐LAMP reactions were carried out using WarmStart™ LAMP 2× Master Mix (DNA & RNA) or WarmStart™ Colorimetric LAMP 2× Master Mix (DNA & RNA) from New England Biolabs (NEB).

A 25 µl reaction mixture (2× MasterMix, 12.5 µl; 10× primer mix, 2.5 µl; DNA/RNA target, 1 µl; DNase & RNase‐free molecular grade water, 9 µl) was mixed homogeneously and centrifuged for 1 s. LAMP or RT‐LAMP was performed in a thermocycler at 65°C for 30 min. Colour change can be observed directly by the naked eye, and gel electrophoresis was performed to confirm the result.

#### LAMP and RT‐LAMP for human cells

All equipment, laminar‐flow cabinet and working bench were sprayed with RNaseZap™ prior to experimental work. Filtered pipette tips were used to prevent cross‐contamination.

Human episomal iPSCs obtained from Thermo Fisher Scientific (UK) were dissociated with 0.5 mM EDTA (pH 8.0; Thermo Fisher Scientific) in sterile PBS. The cells were then counted, resuspended and diluted into 100, 50, and 10 cells µl^−1^ in sterile 0.85% NaCl (Sigma‐Aldrich) solution.

A 10× β‐actin primer mix (FIP, 16 µM; BIP, 16 µM; F3, 2 µM; B3, 2 µM) was made before the reactions, and LAMP and RT‐LAMP were carried out using WarmStart™ Colorimetric LAMP 2× Master Mix (DNA & RNA) from New England Biolabs (NEB). For the one‐step amplification of nucleic acids, cells in 200 µl 0.85% NaCl were first heated at 65°C for 10 min and a 25‐µl reaction mixture (2× MasterMix, 12.5 µl; 10× primer mix, 2.5 µl; the boiled cell sample, 1 µl; DNase & RNase‐free molecular grade water, 9 µl) was prepared. On the other hand, for the cell in LAMP experiments, cells were directly added into the LAMP reaction reagents, where a 25‐µl reaction mixture (2× MasterMix, 12.5 µl; 10× primer mix, 2.5 µl; 0.85% NaCl solution with or without iPSCs , 1 µl; DNase & RNase‐free molecular grade water, 9 µl) was prepared. LAMP/RT‐LAMP was performed in a thermocycler at 65°C for 30 or 40 min. Colour change was observed directly by the naked eye.

### RNA extraction from clinical samples

Respiratory specimens (throat swabs) collected from patients were immediately placed into sterile tubes containing 3 ml of VTM (Health Gene Technologies, Ningbo, China). In total, 16 clinic samples were collected from patients. The swab samples were deactivated by heating at 56°C for 30 min in a biosafety level 2 (BSL 2) medical laboratory of Shenzhen Luohu People's Hospital in China. RNA was extracted from swab samples using RNA extraction kit (Health Biomed, China) on a Smart LabAssist‐32 platform (Taiwan Advanced Nanotech Inc, Taoyuan, China). To avoid the interference of TE buffer to RT‐LAMP reaction, RNA was eluted by RNase‐free and DNase water here.

### Conventional RT‐qPCR for SARS‐CoV‐2

A commercial 2019‐nCoV RT‐PCR kit (Shanghai ZJ Bio‐Tech, China) was used to determine if the samples are positive or negative to SARS‐CoV‐2 virus. in an ABI 7500 Real‐Time PCR System (Thermo Fisher Scientific Inc., Waltham, USA) according to the manufacturer’s instructions. A 25 μl reaction mixture contained 5 μl of RNA as the template, 19 μl of 2019‐nCOV RT ‐PCR Buffer, 1 μl of RT‐qPCR enzyme mix. The thermal cycling condition was 10 min at 45°C for reverse transcription, 3 min at 95°C for PCR initial activation and 45 cycles of 15 s at 95°C and 30 s at 58°C.

### RT‐LAMP for clinical RNA samples

RT‐LAMP assays on clinical samples were performed in pre‐mixed test kits. Each test kit consists of three tubes #1, #2 and #3, which contain O‐117, N‐15 and human β*‐actin* primers respectively (Table 1). The three tubes were first filled up with 5 μl of RNase‐free water (Sigma‐Aldrich), 12.5 μl of WarmStart Colorimetric Lamp 2× Master Mix (New England Biolabs, UK), and 2.5 μl of 10x primer mix. The test kits were first prepared in OSCAR and delivered to Shenzhen Luohu People’s hospital in an ice box. For the detection of SARS‐CoV‐2 virus, 5 μl of extracted RNA from each patient sample was added into tube #1, #2 and #3 respectively. The test kit was incubated at 65°C for 30 min.

## Conflict of interest

None declared.

## Supporting information


**Appendix S1. **Synthesised DNA with T7 promoter.
**Fig. S1. **The RNA map of SARS‐CoV‐2 and the locations of four sets of primers: O‐117, S‐17, N1 and N‐15, which target the regions encoding Orf1ab, Spike protein, and N protein.
**Fig. S2. **The impact of virus transport media to the reading of RT‐LAMP results. All tubes contain N15 primers and WarmStart Colorimetric Lamp Master Mix. The tubes were incubated at 65°C for 30 min.
**Fig. S3. **Real‐time fluorescence RT‐qPCR for clinical samples using the commercial 2019‐nCoV RT‐PCR kit. (A) The reaction amplification curve of the orf1ab gene of 8 positive samples. (B) The reaction amplification curve of the E gene of 8 positive samples. (C) The reaction amplification curve of the N gene of 8 positive samples. (D) The reaction amplification curve of the RNase P gene as internal control of 8 positive samples. (E) The reaction amplification curve of the positive control. (F) The reaction amplification curve of the negative control.
