# Supplementary material for: RT‐LAMP for rapid diagnosis of coronavirus SARS‐CoV‐2
Source: Microb Biotechnol. 2020 Apr 25;13(4):950–61. doi: 10.1111/1751-7915.13586 (PMC7264870; doi:10.1111/1751-7915.13586)
Supplement: Supplementary file 1 — Appendix S1. Synthesised DNA with T7 promoter. Fig. S1. The RNA map of SARS‐CoV‐2 and the locations of four sets of primers: O‐117, S‐17, N1 and N‐15, which target the regions encoding Orf1ab, Spike protein, and N protein. Fig. S2. The impact of virus transport media to the reading of RT‐LAMP results. All tubes contain N15 primers and WarmStart Colorimetric Lamp Master Mix. The tubes were incubated at 65°C for 30 min. Fig. S3. Real‐time fluorescence RT‐qPCR for clinical samples using the commercial 2019‐nCoV RT‐PCR kit. (A) The reaction amplification curve of the orf1ab gene of 8 positive samples. (B) The reaction amplification curve of the E gene of 8 positive samples. (C) The reaction amplification curve of the N gene of 8 positive samples. (D) The reaction amplification curve of the RNase P gene as internal control of 8 positive samples. (E) The reaction amplification curve of the positive control. (F) The reaction amplification curve of the negative control. [file MBT2-13-950-s001.pdf]

Supplementary information

RT-LAMP for rapid diagnosis of coronavirus SARS-CoV-2

Wei E. Huang[1, 2#*], Lim Boon[2#], Chia-Chen Hsu[2,3#], Dan Xiong[4], Wei Wu[4], YeJiong Yu[2,3], Huidong Jia[1], Yun Wang[1], Yida Zeng[1], Mengmeng Ji[1], Hong Chang[1], Xiuming Zhang[4], Hui Wang[1], and Zhanfeng Cui[1,2,3]

[1] Oxford Suzhou Centre for Advanced Research (OSCAR), University of Oxford, Suzhou Industrial Park, Jiangsu, P.R. China.

[2] Department of Engineering Science, University of Oxford, Parks Road, OX1 3PJ, Oxford, United Kingdom.

[3] Institute of Biomedical Engineering, Department of Engineering Science, University of Oxford, OX3 7DQ, United Kingdom.

[4] Medical Laboratory of Shenzhen Luohu People's Hospital,Shenzhen, 518001, Shenzhen,

Synthesised DNA with T7 promoter

N1-T7
CCATGGgtTAATACGACTCACTATAGGGAGATgataatggaccccaaaatcagcgaaatgcaccccgcatt
acgtttggtggaccctcagattcaactggcagtaaccagaatggagaacgcagtggggcgcgatcaaaacaacgtcggcccc
aaggtttacccaataatactgcgtcttggttcaccgctctcactcaacatggcaaggaagaccttaaattccctcgaggacaaggc
gttccaattaacaccaatagcagtccagatggatccact

N15-T7
CCATGGgtTAATACGACTCACTATAGGGAGATgcaactgagggagccttgaatacaccaaaagatcacatt
ggcacccgcaatcctgctaacaatgctgcaatcgtgctacaacttcctcaaggaacaacattgccaaaaggcttctacgcagaa
gggagcagaggcggcagtcaagcctcttctcgttcctcatcacgtagtcgcaacagttcaagaaattcaactccaggcagcagt
aggggaacttctcctgctagaatggctggcaatggcggtgatgcggatccact

S17-T7
CCATGGgtTAATACGACTCACTATAGGGAGAcactaattctttcacacgtggtgtttattaccctgacaaagttttc
agatcctcagttttacattcaactcaggacttgttcttacctttcttttccaatgttacttggttccatgctatacatgtctctgggaccaatg
gtactaagaggtttgataaccctgtcctaccatttaatgatggtgtttattttgcttccactgagaagtctaacataataagaggctgga
tttttggtactactggatccact

O117-T7
CCATGGgtTAATACGACTCACTATAGGGAGAGttacttaccccaaaatgctgttgttaaaatttattgtccagcat
gtcacaattcagaagtaggacctgagcatagtcttgccgaataccataatgaatctggcttgaaaaccattcttcgtaagggtggtc
gcactattgcctttggaggctgtgtgttctcttatgttggttgccataacaagtgtgcctattgggttccacgtgctagcgctaacatagg
atccact

*yellow highlight is the sequence of T7 promoter.

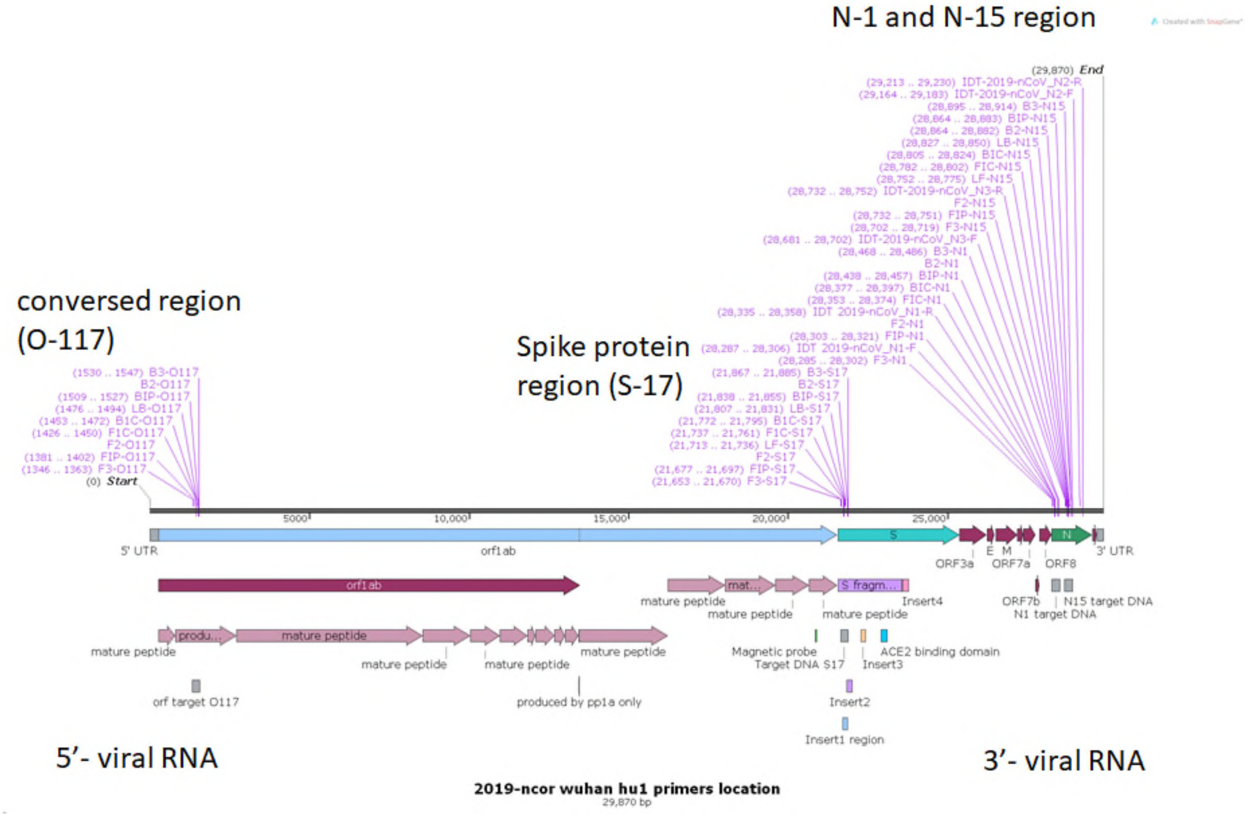

Figure S1. The RNA map of SARS-CoV-2 and the locations of four sets of primers: O-117, S-17, N1 and N-15, which target the regions encoding Orf1ab, Spike protein, and N protein.

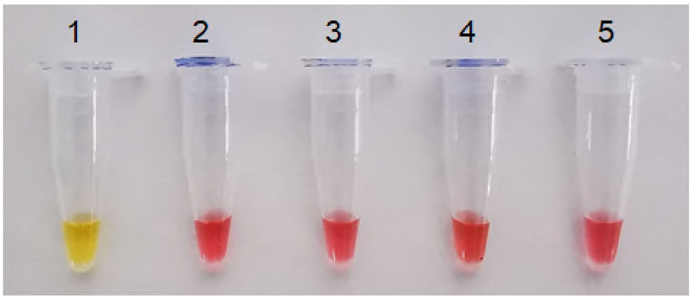

1. 4 µl H_2O + 1 µl N15 DNA fragment
2. 4 µl virus transport media (Yocon Ltd, Beijing, China) + 1 µl N15 DNA fragment
3. 4 µl H_2O + 1 µl human RNA
4. 5 µl virus transport media (Yocon Ltd, Beijing, China)
5. 5 µl H_2O

Figure S2. The impact of virus transport media to the reading of RT-LAMP results. All tubes contain N15 primers and WarmStart Colorimetric Lamp Master Mix. The tubes were incubated at 65 ^{o}C for 30 min.

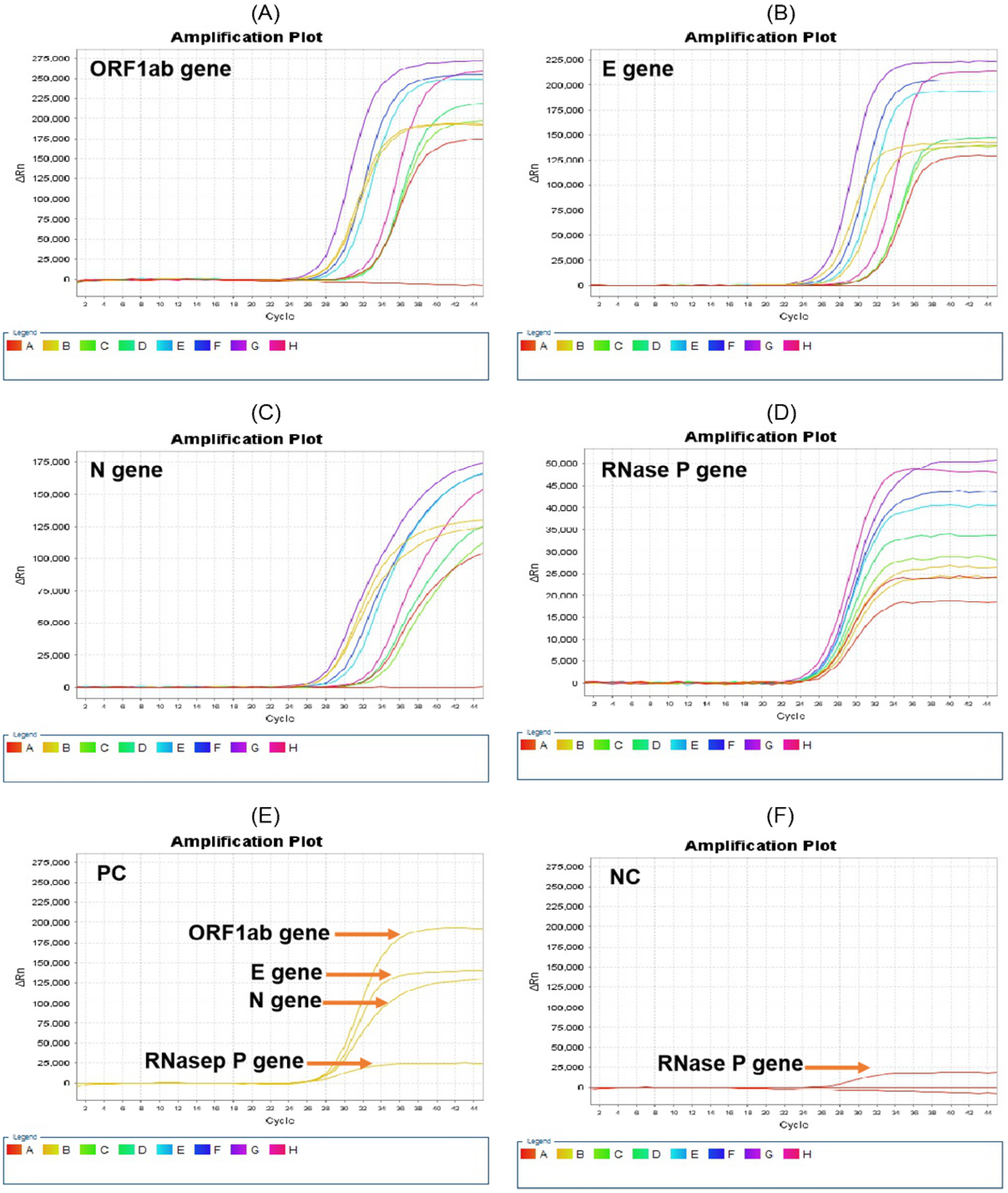

Figure S3. Real-time fluorescence RT-qPCR for clinical samples using the commercial 2019-nCoV RT-PCR kit. (A) The reaction amplification curve of the orf1ab gene of 8 positive samples. (B) The reaction amplification curve of the E gene of 8 positive samples. (C) The reaction amplification curve of the N gene of 8 positive samples. (D) The reaction amplification curve of the RNase P gene as internal control of 8 positive samples. (E) The reaction amplification curve of the positive control. (F) The reaction amplification curve of the negative control.
